# Body Perception Disturbances in Chronic Limb Pain: Exploring Different Assessment Tools and Their Associations with Upper- and Lower-Limb Disability in a Cross-Sectional Study

**DOI:** 10.3390/jcm15103876

**Published:** 2026-05-18

**Authors:** Hana Karpin, Irit Weissman-Fogel, Anatoly Livshitz, Yishai Bachar Kirshenboim, Jean Jacques Vatine

**Affiliations:** 1 Physical Therapy Department, Faculty of Social Welfare and Health Sciences, University of Haifa, Haifa 3103301, Israel; hanakarpin@gmail.com (H.K.); ifogel@univ.haifa.ac.il (I.W.-F.); 2 Reuth Rehabilitation Hospital, Tel Aviv 6772829, Israel; alivshitz2012@gmail.com; 3 Department of Occupational Therapy, School of Health Professions, Sackler Faculty of Medicine, Tel Aviv University, Tel Aviv 6997801, Israel; eshyba@gmail.com; 4 Physical Medicine and Rehabilitation Department, Gray Faculty of Medical & Health Sciences, Tel Aviv University, Tel Aviv 6997801, Israel

**Keywords:** chronic limb pain, body perception disturbances, limb disability, symptom severity, complex regional pain syndrome

## Abstract

**Background**: Body perception disturbances (BPDs) are highly prevalent in chronic limb pain, manifesting as hostile feelings towards the limb, reduced sensory–motor function, and altered limb ownership. Various questionnaires and tests assess BPD, but their interrelation and associations with limb disability remain underexplored. This study aimed to evaluate BPD assessments, examine whether they capture overlapping or distinct aspects of BPD, and determine their associations with upper- and lower-limb disability. **Methods**: This observational cross-sectional study included 92 participants with chronic limb pain. Participants completed BPD questionnaires (Bath-BPD, Neurobehavioral) and tests (Laterality Recognition, Fingers/Toe Perception, Human Figure Drawing), along with clinical pain measures and the symptom severity index. Dependent outcomes were upper- and lower-limb disability. **Results**: Cluster analysis grouped all measures into ‘High’ and ‘Low’ BPD severity clusters, with overall cluster quality rated as fair (silhouette = 0.40). The Neurobehavioral questionnaire emerged as the primary contributor to the cluster structure (silhouette = 1). The ‘High’ BPD cluster showed significantly greater symptom severity (*t*(63) = −4.13, *p* < 0.001). Pain severity, Bath-BPD questionnaire, and symptom severity were significantly associated with upper-limb disability (*p* < 0.001), whereas pain intensity alone accounted for 24% of lower-limb disability variance. **Conclusions**: Despite the diversity of BPD assessments, the clustering pattern suggests a partial convergence between measures, though a weak cohesiveness of BPD tests indicates that they capture partially distinct aspects of body perception. BPD questionnaires were associated with upper-limb disability, whereas lower-limb disability was associated with pain intensity. These findings emphasize the importance of assessing BPDs in musculoskeletal care and suggest their potential role as indicators of symptom severity.

## 1. Introduction

Chronic pain is associated with significant disability, restricted mobility, psychological distress, and reduced quality of life [1]. In chronic limb pain conditions, the perception of the affected limb can become distorted, giving rise to body perception disturbances (BPDs) [2,3]. BPD encompasses altered ownership of the limb, negative or aversive feelings toward it, and disturbances in perceived limb size or position [2], with a reported prevalence of 54–84% [4]. BPD has been associated with higher pain intensity [5,6], impaired limb localization [7], reduced tactile acuity [5], and a poorer quality of life [8,9]. Understanding what underlies these disturbances requires clarity about how BPD is conceptualized and measured.

BPD is typically assessed through self-report questionnaires and performance-based tasks that reflect two components of body representation: body image and body schema. Body image refers to the conscious, attitudinal representation of one’s body, whether perceptual, conceptual, or emotional, whereas body schema reflects the dynamic sensorimotor representation supporting action execution [10]. These systems are considered partially distinct yet interacting components that together guide goal-directed and functional movement [11]. In this framework, self-report questionnaires primarily capture aspects of body image, including perceived distortions, ownership, and affective responses toward the painful body part [12]. In contrast, performance-based tasks such as limb laterality recognition and finger or toe identification are thought to predominantly engage body schema processes, as they rely on implicit sensorimotor representations [10,13]. Limb or body drawing tasks may additionally reflect structural body knowledge, based on a visuospatial body map [14,15], constituting a distinct type of non-semantic body image [10,14].

A key question is whether different BPD assessment tools capture distinct aspects of body representation that manifest as distinct clinical phenotypes or whether they represent different expressions of a shared underlying construct [13,14]. This issue has important clinical implications. If different measures indeed reflect distinct components of body representation, clinicians must determine which therapeutic approach, such as graded motor imagery, mirror therapy, sensorimotor retraining [16], or body image-targeted interventions [17], is most appropriate for the specific BPD identified by these assessments. This may ultimately inform more targeted, mechanism-driven rehabilitation.

However, understanding BPD in isolation is insufficient, as pain and body perception are inherently subjective and interacting experiences [18]. Experimental studies in healthy individuals demonstrate that pain can distort body image [19], while altered body perception can, in turn, modulate pain [20]. In clinical populations, BPD has been associated with specific kinesthetic pain qualities in musculoskeletal conditions [21] and with pain intensity in CRPS [8,22], though the causal direction of these relationships remains unclear [9]. Nevertheless, the relationships between BPD, pain, and disability remain poorly characterized in musculoskeletal disorders [23]. Limited evidence, based on small samples, suggests that BPD may negatively impact upper-limb function [6]. This gap is especially notable given that upper- and lower-limb function differ in their perceptual and functional characteristics, which may influence the manifestation of BPD.

Body representation relies on the integration of vision, touch, and proprioception, with the relative contributions of these modalities varying across body regions [24]. Functionally, the upper limbs are primarily involved in object manipulation, whereas the lower limbs support locomotion and weight-bearing. These differences suggest that BPD may manifest differently and carry distinct functional consequences depending on the limb affected. Yet, this possibility has received little empirical attention.

The present study, therefore, aimed to evaluate a range of BPD measures, examine their sensitivity, explore their clustering and interrelationships, and determine their specific associations with upper- and lower-limb disability. We hypothesized that BPD measures would cluster into distinct profiles reflecting dissociable body representation systems. We further hypothesized that BPD measures would show differential associations with upper- and lower-limb disability.

## 2. Materials and Methods

### 2.1. Participants

All participants were aged >18 years and had limb pain persisting for more than three months following the primary injury. Exclusion criteria included bilateral injury, a primary psychiatric diagnosis, other primary pain syndromes, disorders of the central nervous system, diseases with sensory or inflammatory components, pregnancy or breastfeeding, and severe visual impairment or intellectual disability.

### 2.2. Procedure

This observational study employed a cross-sectional design and is reported in accordance with the Strengthening the Reporting of Observational Studies in Epidemiology (STROBE) guidelines [25]. The institutional review board of Reuth Rehabilitation Hospital approved the study (Approval No. 2017-14). Written informed consent was obtained from all participants prior to enrollment. Participants were recruited through convenience sampling from Reuth pain rehabilitation unit (Tel Aviv, Israel) between June 2018 and January 2021. The present study was conducted as part of a larger assessment battery addressing multiple aims [9], in which participants completed four or five 45–60 min sessions within one month, with sessions scheduled no more than one week apart. For the current study, participants completed two to three of these sessions. In the first session, a pain specialist physician assessed all participants to diagnose complex regional pain syndrome (CRPS) according to the Budapest criteria [26]. Participants who did not meet the Budapest criteria were diagnosed with non-CRPS chronic limb pain. The symptom severity of all participants was evaluated using the CRPS Severity Score (CSS) [27] administered by two pain specialist physicians (J.J.V. and A.L.). Subsequent sessions involved completing BPD tests and questionnaires, as well as evaluating the disability of the Arm, Shoulder, and Hand (DASH), the Lower Extremity Functional Scale (LEFS), and the Short-Form McGill Pain Questionnaire (SF-MPQ). BPD test tasks and questionnaires were administered in separate sessions to minimize cross-task influence (e.g., pain), and the order of test administration was otherwise standardized across participants.

To minimize the effects of fatigue, pain, and attentional load, sessions were paced according to each participant’s symptom severity and attentional capacity, with breaks provided as needed. Self-administered questionnaires were completed in the presence of the first author or a research assistant to clarify unclear items. Assessors were not blinded to participants’ clinical status; while this may have introduced bias, it was necessary to accommodate symptom fluctuations across sessions.

### 2.3. Measures

#### 2.3.1. BPD Measures

These measures were selected based on their prior use in CRPS and chronic limb pain studies, supporting their clinical relevance and applicability within this population [4].

##### Body Perception Self-Report Questionnaires

These self-report questionnaires are considered to assess body image, reflecting conscious perceptual, cognitive, and affective aspects of body representation [10]. The Bath Body Perception Disturbances and Neurobehavioral questionnaires were administered in Hebrew using standard Hebrew versions. Convergent validity [28] between the measures was supported by a significant correlation (*r* = 0.61, *n* = 90, *p* < 0.001).

The Bath-BPD Questionnaire [2] includes seven items regarding aspects related to the perceptiveness of the affected limb: ownership, limb position awareness, attention to the painful limb, the feelings towards it, perceptual disparities in size, temperature, pressure, and weight; limb amputation desire; and a mental representation of the affected limb. The total score ranges from 0 to 57 points [2].

The Neurobehavioral questionnaire [29] contains five items: two assess the presence of motor neglect symptoms (‘If I do not focus my attention on my painful limb, it would lie still, like dead weight’ and ‘I need to focus all of my attention on my painful limb to make it move the way I want it to), and two assess the presence of cognitive neglect (‘My painful limb feels as though it is not part of my body’ and ‘My painful limb feels dead to me’). The fifth item assesses the presence of involuntary movements. The expected answer is dichotomous (true or false). The test’s scoring method was expanded to a 6-point Likert scale (1 = never, 6 = always) to indicate the severity of the neglect symptoms [30]. This adapted questionnaire was used in the current study, calculated as the sum of the five items. In the current study, both the original dichotomous scoring and the adapted Likert scoring were applied, showing a high and significant correlation (*r* = 0.88, *p* < 0.001), supporting the validity of the adapted scoring approach.

##### Body Perception Tests

Human Figure Drawing test: Healthy controls were included only in a predefined exploratory sub-study to evaluate the discriminative sensitivity of the novel Human Figure Drawing test and should be interpreted as a methodological validation component rather than part of the primary study design. The other BPD measures were not compared with healthy controls in the current study, as their discriminative validity has been previously established for the Bath-BPD [6]; neurobehavioral differentiation from other pain conditions [30,31]; laterality recognition, with differences between the affected and unaffected sides in CRPS demonstrated [32]; and the finger/toe perception test, with side-to-side differences also demonstrated [33].

Participants were instructed to draw a single human figure (male or female, at their discretion) on a blank white A4 sheet. All drawings were digitized and analyzed using ImageJ software (https://imagej.net, accessed on 15 January 2021) [34]. To maintain objectivity in the test scoring, the figure size (the outline circumference of the drawn figure in cm) and the angle size—the slope angle formed between the feet and the horizontal baseline—were measured. For comparison, 29 drawings from a convenience sample of age-matched healthy controls (mean age 36.6 ± 9.2; 13 males, 15 females) were analyzed using the same criteria. Healthy controls were adults (>18 years) with no history of chronic pain, neurological or psychiatric conditions, or limb injury affecting function, and no current pain complaints. The test was included as an exploratory measure of non-semantic body image reflecting structural body knowledge [14]. Drawing-based methods have been shown to capture distortions in body representation in chronic pain conditions, including CRPS [35], although variability in scoring methods has been noted [36]. In the present study, a quantitative and objective scoring approach was applied. Given its indirect nature, these indices were interpreted cautiously as non-specific indicators of body representation rather than direct measures of BPD.

Fingers/Toes Perception test: This test assesses the ability to identify fingers and toes using mechanical stimulation with vision occluded [33], relying on implicit tactile processing and thereby reflecting body schema and somatosensory representation [10,14]. The examiner touched the distal interphalangeal joint of each finger or toe with a cotton pad twice in a uniform order (10 stimuli per hand/foot). Subjects were required to name the finger or toe they felt was being touched. The test was performed on both limbs, starting with the non-painful limb.

Laterality Recognition test: We used the Recognize™ app (Neuro Orthopedic Institute, Adelaide, Australia). The test involved 30 images of hands/feet shown in different positions for 10 s each on a tablet. Participants pressed “Rt” for right-side images and “Lt” for left-side images. Each test was repeated three times. Hit rates (percentages) and reaction times (seconds) were recorded separately for each body side and averaged over the three tests. Results were analyzed by comparing painful and non-painful limbs. This task was included as a measure of body schema—the dynamic sensorimotor representation of one’s body derived from sensory input and integrated with motor systems [32].

#### 2.3.2. Symptom Severity Evaluation

##### CRPS Severity Score (CSS) [27]

Complex regional pain syndrome (CRPS) is a chronic pain condition, usually affecting one limb after trauma. It is marked by extreme pain disproportionate to the injury, accompanied by fluctuating sensory, autonomic, motor, and trophic disturbances.

The CSS is a quantitative index that contains 16 CRPS signs and symptoms in 4 categories: sensory, vasomotor, sudomotor/edema, and motor/trophic criteria. Each presence sign during medical assessment or symptom reported by the subject is coded 1/0 and summed up to create an overall CRPS Severity Score (0–16). A higher score indicates a more severe CRPS syndrome. The CSS was used as an index of symptom severity. In the present study, it was applied as a continuous measure of symptom severity across the entire sample, reflecting shared clinical features between CRPS and non-CRPS limb pain conditions [37].

#### 2.3.3. Pain Severity Evaluation

##### Short-Form McGill Pain Questionnaire (SF-MPQ) [38]

It is a self-report measure that assesses the quality and intensity of clinical pain. It includes 11 sensory and four emotional descriptions rated on a 0–3 scale (no, mild, moderate, strong). The total score ranges from 0 to 45, with higher scores indicating more severe pain. Additionally, it includes the verbal pain bar from 0 (“no pain at all”) to 5 (“extreme pain”) and the Visual Analogue Scale (VAS), a 100 mm line from “no pain” to “maximal pain imaginable,” which was used in the current study as a measure of pain intensity.

#### 2.3.4. Limb Disability Evaluation

##### The Disabilities of the Arm, Shoulder, and Hand Questionnaire (DASH) [39]

This is a self-report questionnaire developed to measure physical disability and symptoms among people with a musculoskeletal disability affecting the upper limb. It includes 30 items, coded on a scale ranging from 1 (no difficulty) to 5 (unable to perform the activity). 24 items refer to the disability in performing the activity, and six items refer to the severity of the symptoms (pain, activity-related pain, stiffness, tingling, and weakness). The score related to the limitation/symptom questionnaire ranges from 0 to 100, whereas higher scores indicate more severe disability.

##### The Lower Extremity Functional Scale (LEFS) [40]

This is a self-report questionnaire examining the effect of musculoskeletal impairment on the ability to perform functional activities related to the lower limbs (e.g., walking, squatting, getting out of a car, climbing stairs). The questionnaire comprises 20 items, each coded on a scale ranging from 0 (inability to perform) to 4 (performance without difficulty). The final score ranges from 0 to 80 points and is obtained by summing up all the questionnaire items. The lower the score, the more severe the functional disability.

### 2.4. Statistical Analysis

All statistical analyses were conducted using IBM SPSS Statistics version 27. Data are presented as mean ± SD for continuous variables and as frequencies and percentages for categorical variables. Statistical significance was defined as *p* ≤ 0.05. Effect size was calculated as Cohen’s *d* [41], with values of 0.20, 0.50, and 0.80 indicating small, medium, and large effects, respectively. We evaluated differences between the painful and nonpainful limbs on the Finger/Toe Perception and Laterality Recognition tests using paired *t*-tests. An independent-samples *t*-test was used to examine differences in symptom severity between the high- and low-BPD cluster groups.

To address the study aim, we used cluster analysis to examine how BPD assessments group together based on shared variance, allowing us to determine whether they capture overlapping or distinct dimensions of BPD. A two-step cluster procedure (pre-clustering followed by hierarchical clustering) automatically identified the optimal cluster structure, with silhouette coefficients indicating cluster quality [42]. Prior to analysis, all clustering variables were z-transformed. Associations with limb involvement and injury side were examined using χ^2^ tests and eta-squared.

Pearson correlations were used to evaluate the relationships among BPD measures, pain, symptom severity, and limb disability. Correlations were conducted using pairwise complete cases: Bath-BPD (*n* = 30), Neurobehavioral questionnaire (*n* = 31), SF-MPQ total (n = 31), MPQ-VAS (*n* = 31), and CSS (*n* = 29). Given the limited sample size available for upper-limb analyses (*n* = 31), bivariate correlations are reported rather than regression. For lower-limb disability, a regression analysis using the Enter method was conducted (*n* = 52).

Sample size and statistical power: Since no definitive methods exist for calculating a priori power in cluster analysis [43], we followed guidance suggesting a minimum sample size of N = 20 per subgroup for a large cluster separation (Δ = 4) [43].

Missing data: Several measures had missing data: (i) in the Finger/Toe Perception test, 79 participants completed the assessment, with missing data primarily due to severe allodynia (i.e., tactile hypersensitivity) in the painful limb; (ii) in the Human Figure Drawing test, 79 participants completed the task, as some participants were enrolled before the test was added to the protocol; (iii) one participant did not complete the Bath-BPD questionnaire; (iv) the CSS was administered to 87 participants, with missing data for 5 participants. The cluster analysis required complete data across all included variables; therefore, only participants with no missing data on any clustering variable were included, resulting in a final sample of *n* = 68. Of the 36 participants with upper-limb involvement, 5 did not complete the DASH. Of the 56 participants with lower-limb involvement, 4 did not complete the LEFS (final regression sample: *n* = 52).

## 3. Results

### 3.1. Participants

**Figure 1:**
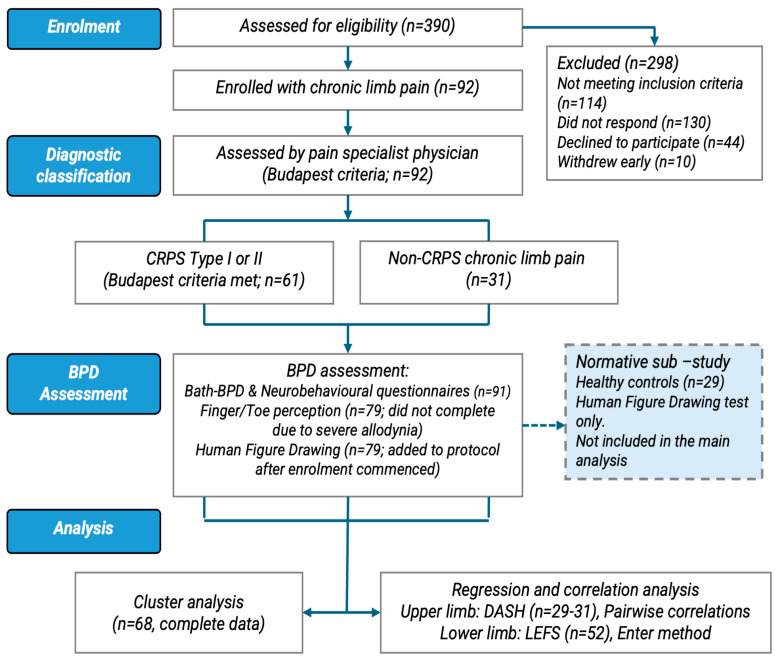
Flow diagram of participant recruitment, eligibility, diagnostic classification, and analysis.

390 patients were assessed for eligibility. Of these, 298 were excluded (114 did not meet the inclusion criteria, 130 did not respond, 44 declined participation, and 10 withdrew early). The final sample comprised 92 adults with chronic limb pain after traumatic injury, of whom 61 met the Budapest criteria for CRPS type I or II [44], and 31 did not meet this threshold and were classified as non-CRPS, chronic limb pain (see Figure 1).

There were no significant differences between groups in age, disease duration, or etiology distribution (*p* > 0.05). Disease duration was highly variable across the full sample (*M* = 744.52 days, SD = 879.68), reflecting a wide range of chronicity that may have introduced heterogeneity in BPD severity and disability outcomes. The sex distribution differed significantly between groups, with a higher proportion of females in the non-CRPS group (74.2%) compared to the CRPS group (52.5%; *p* = 0.044); this imbalance was accounted for in regression analyses (see Section 3.7). Limb involvement was distributed across upper (*n* = 31) and lower limbs (*n* = 52), with a similar distribution across diagnostic groups (*p* = 0.953). No significant differences between groups were found in age, education, disease duration, injury type, or side of involvement (all *p* > 0.05). Full demographic characteristics are presented in Table 1.

### 3.2. Descriptive and Discriminative Statistics of BPD Tools

Table 2 presents within-subject comparisons between painful and non-painful limbs (Laterality Recognition and Finger/Toe Perception tests) and descriptive statistics for the BPD questionnaires. The Human Figure Drawing normative comparison is presented separately in Table 3. Measures differed markedly in their ability to distinguish between the painful and non-painful limb. Using paired *t*-tests, the Laterality Recognition test showed no significant differences in hit rates or reaction times between limbs (*p* > 0.05), indicating that this task did not capture limb-specific perceptual disturbances in this sample. By contrast, in the Finger/Toe Perception test, participants correctly identified significantly fewer fingers/toes on the painful limb compared to the non-painful limb across total score, toes, and fingers (all *p* < 0.05). Effect sizes ranged from small to medium for the total score (*d* = 0.26) and toes (*d* = 0.43) and from medium to large for finger identification (*d* = 0.64). The Human Figure Drawing test revealed that participants with chronic pain drew significantly smaller figures and larger angles compared to healthy controls, with a large effect for image size (*d* = 0.85) and a medium effect for angle size (*d* = 0.52). The angle size, reflecting laterality differences between body sides, was included in the cluster model to align with the study’s focus on participants experiencing chronic, asymmetric regional limb pain.

### 3.3. The BPD Cluster Analysis Model

A two-step cluster analysis was conducted to identify patterns across BPD measures [45]. This method was selected for its ability to minimize the influence of dominant variables and to estimate each measure’s relative contribution to the cluster solution.

Cluster Description: The model included the Neurobehavioral and Bath Body Perception questionnaires, the Fingers/Toes Perception test on the painful side, and the Human Figure Drawing test (angle size), selected based on their discriminative validity and theoretical relevance. Notably, the laterality recognition test was excluded from the cluster model because it failed to discriminate between the painful and non-painful sides and, therefore, did not contribute to the subsequent analysis.

The number of clusters was determined automatically by the algorithm using Schwarz’s Bayesian Information Criterion (BIC) [46]. Given the heterogeneity of BPD measures, the analysis was considered exploratory. Model fit was evaluated using the average silhouette coefficient, an internal validity index that reflects cluster cohesion and separation (range: 0–1), with higher values indicating better fit [42]. Cluster composition (CRPS vs. non-CRPS) was described as frequencies and percentages, and associations with clinical characteristics (CRPS severity, sex, affected region, and side of injury) were examined using chi-square tests.

**Figure 2:**
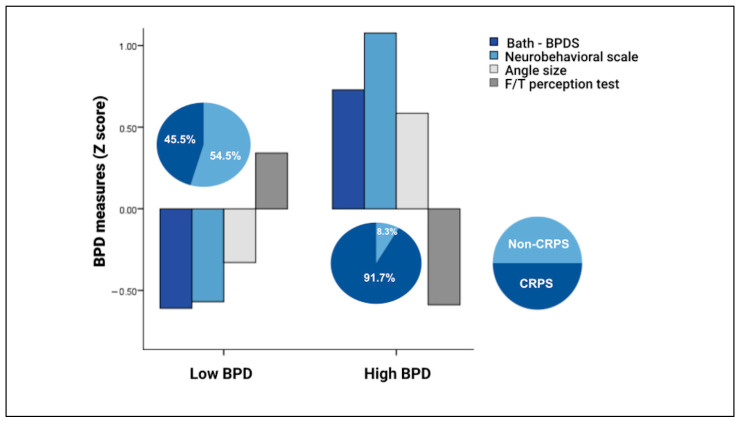
High and Low BPD clusters. The bar chart presents Z-scores for each BPD measure within the Low and High BPD clusters. The pie charts indicate the distribution of clinical groups (CRPS and non-CRPS) in each cluster. For the Bath-BPD, Neurobehavioral, and angle size measures, higher scores indicate greater BPD; for the Finger/Toe Perception test, lower scores indicate greater BPD. Note. CRPS = complex regional pain syndrome; CLP = chronic limb pain; Bath-BPD = Bath Body Perception Disturbances Questionnaire; Neurobehavioral = Neurobehavioral questionnaire; F/TP = Finger/Toe Perception test.

Two clusters emerged, representing low BPD (‘Low’, *n* = 44) and high BPD (‘High’, *n* = 24), with each cluster comprising all body perception measures (see Figure 2). The Low cluster included 20 CRPS participants (45.5%) and 24 non-CRPS participants (54.5%), whereas the High cluster comprised 22 CRPS participants (91.7%) and 2 non-CRPS participants (8.3%) (see Figure 2).

### 3.4. Cluster Validation

Cluster Quality: Overall cluster quality was fair (silhouette = 0.40). Cohesion was highest for the Neurobehavioral and Bath-BPD questionnaires (silhouette = 1 and 0.59, respectively), indicating a greater similarity of these measures to the derived cluster. The Fingers/Toes Perception test (silhouette = 0.35) and the Human Figure Drawing (angle size; silhouette = 0.25) demonstrated lower cohesion values, suggesting relatively weaker clustering contributions.

### 3.5. Associations with Disease Characteristics

An independent-samples *t*-test revealed a significant difference in CRPS Severity Scores between the two clusters. The low BPD cluster (*M* = 8.66, SD = 3.30, *n* = 42) showed significantly lower scores compared to the high BPD cluster (*M* = 12.00, SD = 2.69, *n* = 23), *t* (63) = − 4.13, *p* < 0.001.

Cluster membership was significantly correlated with the original diagnosis (CRPS vs. non-CRPS) (Eta = 0.454, *p* < 0.001) but showed no significant association with sex (χ^2^(1) = 2.01, *p* = 0.123), the affected region (upper vs. lower limb; (χ^2^(1) = 2.584, *p* = 0.089) or the injured side (right vs. left limb); (χ^2^ (1) = 0.472, *p* = 0.333).

### 3.6. Correlation of BPD Tools with Upper- and Lower-Limb Disability

Among participants with upper-limb involvement, the mean DASH score was 53.45 (SD = 23.36), indicating moderate-to-severe upper-limb disability. Among those with lower-limb involvement, the mean LEFS score was 27.75 (SD = 12.38), reflecting substantial functional limitations. The Bath-BPD and Neurobehavioral questionnaires showed a highly significant correlation with the DASH (*r* = 0.67 and 0.64, respectively, *p* < 0.001), whereas the correlations with the LEFS were not significant. The correlations between the finger/toe perception and angle size tests with the DASH and the LEFS were not significant (see Table 4).

### 3.7. Correlation of Clinical Pain and Symptom Severity with Upper and Lower Limb Disability

Pain measures (SF-MPQ Total and VAS) were significantly associated with both upper- and lower-limb disability (all *p* < 0.001). Symptom severity (CSS) was significantly associated with upper-limb disability (*r* = 0.74, *p* < 0.001) but not with lower-limb disability (see Table 3).

### 3.8. Regression Models: Association of BPD, Pain, and Symptom Severity with Lower-Limb Function

Lower Limb: Due to multicollinearity between SF-MPQ total score and VAS (*r* = 0.75, *p* < 0.001), only VAS was included, as it showed the strongest bivariate correlation with LEFS. The model was conducted using the Enter method. VAS alone accounted for 24% of the variance in LEFS scores (*β* = −0.48, *p* < 0.001, 95% CI [−4.28, −1.39]). Given the sex imbalance across groups, all regression analyses were adjusted for sex to account for potential confounding (see Table 5).

## 4. Discussion

This study examined the sensitivity, interrelationships, and functional relevance of different BPD measures in chronic limb pain. Several key findings emerged. First, self-report BPD measures (Neurobehavioral, Bath-BPD) were most strongly associated with upper-limb disability, whereas BPD tests showed limited associations with functional outcomes. Second, the cluster analysis suggested partial convergence across measures, but with only moderate overall quality and uneven contributions across variables. Third, cluster membership was associated with symptom severity, supporting a link between BPD and clinical status. Finally, a differential pattern was observed between limbs: upper-limb disability was associated with BPD, whereas lower-limb disability was primarily related to pain intensity.

The first hypothesis was partially supported: BPD measures clustered into two distinct profiles, with convergence driven primarily by self-report BPD questionnaires, whereas BPD tests contributed more weakly.

The use of different BPD tools raises questions regarding whether they assess different or overlapping clinical phenotypes. The Bath and Neurobehavioral questionnaires assess similar content [22]: attitudes, beliefs, and perceptions toward the painful limb. Accordingly, this overlap was reflected in the clustering results, where these measures were the primary contributors to the cluster structure. In contrast, finger/toe identification and the Human Figure Drawing rely on dynamic somatosensory and topological representations, considered part of the visuospatial aspect of body perception [47]. These tests rely on structural knowledge of body boundaries, proximity, and position based on vision and somatic perception [10]. In line with the triadic framework of body representation [14], the BPD measures tap into different components of body perception: affective–cognitive aspects (body image) captured by the Bath-BPD and Neurobehavioral questionnaires; visuospatial representations of body structure (body structural description) reflected in the Human Figure Drawing; and sensorimotor processes related to limb positioning (body schema), assessed by the Finger/Toe Perception test. The dominance of self-report measures may be theoretically explained by the asymmetric co-construction model [11], which posits that body image can recalibrate body schema when discrepancy exceeds a critical threshold. In chronic limb pain, pain-related sensory hypersensitivity [48] may distort sensorimotor feedback to a degree that exceeds this threshold, rendering body image the clinically dominant representation. This may explain why BPD tests contributed less consistently and weakly to the cluster structure. However, these findings should be interpreted cautiously, given the exploratory nature of the model and its moderate overall quality (silhouette = 0.40). Replication in larger samples using confirmatory methods is needed.

The clinical relevance of the BPD measures is further supported by prior work: a medium effect size was reported for the Neurobehavioral questionnaire in distinguishing CRPS from other chronic limb pain conditions (*d* = 0.57) [30], and the Bath-BPD demonstrated sensitivity to clinically meaningful change following a visual illusion intervention (ES = 0.6) [17]. For the BPD tests, the effect sizes observed between the painful and non-painful limb for the Finger/Toe Perception test (*d* = 0.26–0.64) and Human Figure Drawing compared to healthy controls (*d* = 0.52–0.85) provide novel quantitative reference points, as comparable data have not previously been reported. Established clinical thresholds for these measures remain to be determined, representing an important direction for future research.

The absence of discrimination between limbs on the Laterality Recognition test is consistent with some previous findings [49,50], though it contradicts others [32,51]. These discrepancies may reflect differences in sampling and study design: most prior studies compared pain versus pain-free groups rather than within-participant limb comparisons. Furthermore, Schwoebel et al. (2001) [51] found that limb differences emerged only for large-amplitude imagined movements, a factor not controlled in the current study.

Examining the cluster distribution, the high-severity BPD cluster comprised 91.7% CRPS patients, whereas the low-severity BPD cluster comprised 54.5% non-CRPS patients. While this suggests a strong overall association between CRPS and elevated BPD severity, the presence of CRPS patients in both clusters indicates that BPD disturbance may reflect symptom burden rather than diagnosis per se, pointing to meaningful heterogeneity within the CRPS population. The higher BPD severity observed in CRPS may be explained by several factors. The negative appearance of the painful limb (e.g., edema, color changes, swelling) [52] is more prominent in more severe pain conditions (i.e., CRPS), which may contribute to a more negative body image. Additionally, continuous inescapable pain can lead to maladaptive coping strategies, such as depersonalization and limb detachment, which are also associated with negative body image [9,12]. A negative body image can worsen symptom severity by affecting emotional state (distress and perceived physical impairment) and behavioral coping strategies (catastrophizing and avoidance) [53]. This suggests a vicious cycle in which negative body image, higher pain, and symptom severity reinforce each other and negatively affect function and participation.

Turning to the second hypothesis, this was partially supported: self-report BPD measures were associated with upper- but not lower-limb disability, while BPD tests were not significantly associated with disability in either limb.

The Bath questionnaire captures a wide range of symptoms from the person’s perspective [12], including discrepancies between the limb’s appearance and perception (e.g., larger or warmer), negative feelings towards the limb, and distortions in the limb’s mental image. The Neurobehavioral questionnaire captures cognitive and motor neglect symptoms. Together, these results suggest that both aspects are associated with upper-limb disability. However, the LEFS scale was associated solely with pain intensity. A possible explanation, although not directly tested in the current research, concerns differences in “visual exposure” between the hands and the legs. The hands are seen from many angles, almost always visible, while the legs are mainly seen from a top-down view (i.e., looking down when standing or sitting) [24]. Since body image is primarily based on vision [10], continuously observing the painful hand and its negative appearance may foster a negative body image [20] and reinforce learned non-use processes [54], thereby compounding upper-limb disability beyond what pain alone would be associated with. This hypothesis is further supported by the hand’s greater functional specialization, which is visually guided, its central role in environmental and social interaction, and its larger cortical somatosensory representation compared to the lower limb [55,56,57]. These properties may increase its sensitivity to disturbances in body perception. In line with this, a CRPS study found that improving the visual appearance of the affected hand was associated with better Bath-BPD scores, suggesting a link between visual stimuli and body perception [17]. It should be noted, however, that the DASH contains a small number of pain-related items; thus, a partial overlap with pain measures cannot be fully excluded and should be considered when interpreting the observed associations.

In contrast, lower-limb function is primarily organized around stance, balance, and locomotion [56], where the direct mechanical demands of load and pain during movement may be more proximal drivers of disability independently of BPD. This may explain why pain intensity alone was associated with LEFS scores in the current sample, while body perception measures did not contribute significantly. Other factors, such as kinesiophobia [58] or pain catastrophizing [59], may also contribute to lower-limb disability in chronic pain, which were not tested in the current study and warrant exploration in future studies. Results suggest that body image may be differentially associated with upper- and lower-limb function, warranting further investigation in future studies.

### Strengths and Limitations

The study has several limitations. The study sample was based on patients hospitalized in a pain rehabilitation unit, which may not represent less severe chronic limb pain populations, introducing potential selection bias. The cluster solution should be interpreted with caution, as missing data in two BPD tests were present, with non-random missingness in the Finger/Toes Perception test due to severe allodynia, and overall cluster quality was only moderate. Measurement limitations include the use of a convenience control sample for the Human Figure Drawing test. In addition, the absence of psychological and behavioral measures limits the interpretation of the associations between BPD, pain, and disability. Methodological limitations should also be considered. The sample was heterogeneous (CRPS and non-CRPS; upper and lower limbs), which may introduce residual confounding despite the absence of significant group differences. Furthermore, differences in statistical approaches between upper- and lower-limb analyses limit direct comparability, and the relatively small sample size, particularly in subgroup analyses, may further limit generalizability. Finally, the use of a cross-sectional design precludes causal inferences.

The study has several prominent strengths: To the best of our knowledge, this is the first study to combine a comprehensive battery of BPD questionnaires and tests in chronic limb pain, enabling the assessment of multiple dimensions of body representation within the same sample and examination of their sensitivity and clustering patterns. It is also the first study to examine the association between BPD and lower-limb function. The inclusion of symptom severity allowed the examination of BPD in relation to clinical state, and the direct comparison of upper- and lower-limb disability within the same cohort enabled a more nuanced examination of limb-specific associations with BPD. Finally, the study employed a novel and objective quantitative scoring approach for the Human Figure Drawing test, providing a replicable method for capturing structural aspects of body representation that can be adopted in future studies.

## 5. Conclusions

The present findings carry several clinical implications. The Bath-BPD and the Neurobehavioral questionnaires emerged as the most clinically informative measures, with the strongest association with upper-limb disability, suggesting they capture functionally relevant aspects of BPD in upper-limb conditions. BPD tests, while not independently associated with disability outcomes, may provide complementary and more objective information about distinct aspects of body representation.

Combining self-report and performance-based BPD measures may provide a broader picture of BPD across the affective–cognitive, visuospatial, and sensorimotor dimensions. Future studies should examine whether this combined assessment approach supports more precise, mechanism-informed treatment in chronic limb pain rehabilitation. In addition, further studies are needed to develop and validate a multidimensional model of factors contributing to BPD in chronic limb pain.

## Figures and Tables

**Table 1:** Participants’ demographic characteristics.

Variables	CRPS *n* = 61 Mean (SD)	Non-CRPS *n* = 31 Mean (SD)	*p*	Total Sample *n* = 92 Mean (SD)
Age (years)	34.7 (11.52)	39.0 (15.09)	0.164	36.1 (12.91)
Sex (females/males)	32/29 (52.5/47.5)	23/8 (74.2/25.8)	0.044	55/37 (59.8/40.2)
Education (years)	12.93 (2.14)	13.71 (2.25)	0.111	13.20 (2.2)
Disease duration (days)	728.62 (903.96)	775.81 (843.25)	0.809	744.52 (879.68)
Type of injury				
Fracture	31(50.81%)	14(45.16%)	0.739	45
Soft tissue trauma	27 (44.26%)	15 (48.38%)		42
Other	3 (4.91%)	2 (6.45%)		5
Limb involved				
Upper limb	24 (39.35%)	12 (38.7%)	0.953	36
Lower limb	37 (60.65%)	19 (61.29%)		56
Side involved				
Right	31 (50.81%)	12 (38.71%)	0.715	43
Left	30 (49.19%)	19 (61.29%)		49

Note. CRPS = complex regional pain syndrome; Other = inflammation, acute disease, spontaneous onset.

**Table 2:** Within-subject comparisons between painful and non-painful limbs and descriptive statistics for the BPD questionnaires.

Test	Mean (SD)	Mean (SD)	*T*	*p*	Cohen’s *d*
Laterality Recognition	Painful Limb *n* = 83	Non-Painful Limb *n* = 83			
Time (seconds)	2.56 (1.06)	2.52 (1.08)	*t* (82) = 0.441	0.660	0.05
Recognition (%)	81.27 (15.75)	79.52 (14.83)	*t* (82) = 1.25	0.216	0.14
Fingers/Toes Perception Test	Painful Limb *n* = 79	Non-Painful Limb *n* = 87			
Total (number correct)	7.91 (2.14)	8.39 (1.42)	*t* (78) = 3.42	0.001	0.26
Toes (number correct)	7.53 (1.82)	8.27 (1.61)	*t* (44) = 2.12	0.040	0.43
Fingers (number correct)	8.41 (2.45)	9.59 (0.78)	*t* (33) = 2.73	0.010	0.64
	Chronic pain group *n* = 79	Healthy Group *n* = 29	*T*	*p*	Cohen’s *d*
BPD Questionnaires	Chronic pain group *n* = 91				
Bath-BPD	21.56 (10.99)				
Neurobehavioral	8.43 (5.92)				

**Table 3:** Comparison of Human Figure Drawing indices between chronic pain patients and healthy controls (normative sub-study).

Test	Mean (SD)	Mean (SD)	*T*	*p*	Cohen’s *d*
Human Figure Drawing Test					
Image size (cm)	60.76 (25.11)	82.91 (26.96)	*t* (106) = 3.98	<0.001	0.85
Angle size (°)	5.83 (4.87)	3.83 (2.28)	*t* (105) = 2.41	0.018	0.52

Note. Angle size was measured by the slope angle formed between the feet and the horizontal baseline. number = number of fingers correctly identified. BPD = body perception disturbance.

**Table 4:** Correlations of BPD measures, clinical pain, and symptom severity with upper- and lower-limb disability.

Measure	DASH (Upper Limb)	LEFS (Lower Limb)
BPD Questionnaires		
Bath-BPD	**0.67**	−0.33
Neurobehavioral	**0.64**	−0.19
BPD Tests		
Finger/Toe Perception	−0.05	−0.20
Angle Size	0.37	0.07
Clinical Pain/Symptoms		
SF-MPQ Total	**0.72**	**−0.48**
MPQ-VAS	**0.74**	**−0.49**
CSS	**0.74**	−0.35

Note. Bold values indicate statistical significance following Bonferroni correction (*p* < 0.007). DASH = Disabilities of the Arm, Shoulder, and Hand; LEFS = Lower Extremity Functional Scale; SF-MPQ = Short-Form McGill Pain Questionnaire; VAS = Visual Analog Scale; CSS = CRPS Severity Score.

**Table 5:** Summary of regression analysis for lower-limb disability.

		*R*	*R* ^2^	*R*^2^ Changes	*F* Changes	Significant *F* Changes	*B*	Beta	*t*	*p*	VIF
	LEFS										
1	SF-MPQ-VAS	0.48	0.24	0.24	15.64	<0.001	−3.55	−0.48	−3.95	<0.001	1

LEFS = Lower Extremity Functional Scale; SF-MPQ VAS = Short-Form McGill Pain Questionnaire Visual Analog Scale. B = unstandardized regression coefficient; Beta = standardized regression coefficient; VIF = variance inflation factor.

## Data Availability

The datasets generated and analyzed during the current study are available from the corresponding author upon reasonable request.

## Abbreviations

The following abbreviations are used in this manuscript:

BPDs	Body perception disturbances
CRPS	Complex regional pain syndrome
CSS	CRPS Severity Score
LEFS	Lower Extremity Functional Scale
DASH	Disabilities of the Arm, Shoulder, and Hand Questionnaire
